# Evaluation of two counterflow traps for testing behaviour-mediating compounds for the malaria vector *Anopheles gambiae s.s. *under semi-field conditions in Tanzania

**DOI:** 10.1186/1475-2875-7-230

**Published:** 2008-11-03

**Authors:** Wolfgang H Schmied, Willem Takken, Gerry F Killeen, Bart GJ Knols, Renate C Smallegange

**Affiliations:** 1 Center of Ecology, Faculty of Life Sciences, University of Vienna, Althanstraße 14, 1090 Vienna, Austria; 2 Entomology Unit, Ifakara Health Research and Development Center, Ifakara, Tanzania; 3 Laboratory of Entomology, Wageningen University and Research Center, P.O. Box 8031, 6700 EH Wageningen, The Netherlands; 4 School of Biological and Biomedical Sciences, South Road, Durham, DH1 3LE, UK

## Abstract

**Background:**

Evaluation of mosquito responses towards different trap-bait combinations in field trials is a time-consuming process that can be shortened by experiments in contained semi-field systems. Possible use of the BG Sentinel (BGS) trap to sample *Anopheles gambiae s.s. *was evaluated. The efficiency of this trap was compared with that of the Mosquito Magnet-X (MM-X) trap, when baited with foot odour alone or combinations of foot odour with carbon dioxide (CO_2_) or lemongrass as behaviour-modifying cues.

**Methods:**

Female *An. gambiae s.s. *were released in an experimental flight arena that was placed in a semi-field system and left overnight. Catch rates for the MM-X and BGS traps were recorded. Data were analysed by fitting a generalized linear model to the (n+1) transformed catches.

**Results:**

Both types of traps successfully captured mosquitoes with all odour cues used. When the BGS trap was tested against the MM-X trap in a choice assay with foot odour as bait, the BGS trap caught about three times as many mosquitoes as the MM-X trap (P = 0.002). Adding CO_2 _(500 ml/min) to foot odour increased the number of mosquitoes caught by 268% for the MM-X (P < 0.001) and 34% (P = 0.051) for the BGS trap, compared to foot odour alone. When lemongrass leaves were added to foot odour, mosquito catches were reduced by 39% (BGS, P < 0.001) and 38% (MM-X, P = 0.353), respectively.

**Conclusion:**

The BGS trap shows high potential for field trials due to its simple construction and high catch rate when baited with human foot odour only. However, for rapid screening of different baits in a contained semi-field system, the superior discriminatory power of the MM-X trap is advantageous.

## Background

Due to the role of mosquitoes in disease transmission and their impact on human well-being through their biting behaviour, both commercial and scientific interest exists for efficient trapping devices. During the last century, a number of different mosquito traps and collection methods were developed (reviewed by [1] and [2]). Recently, variations of the CDC light trap [3], the OBET [4,5] and Mbita trap [6-8], electric nets [9] and different traps featuring counterflow geometry [10-14], have been used to evaluate the attractiveness of various complex host odours, individual volatile organic compounds, or mixtures thereof.

While full body odour was often successfully used to attract mosquitoes [15-17], synthetic baits were developed to improve ease of use and to allow standardizing the attractant. As mosquito host-seeking behaviour is governed by semiochemicals, baits can contain a number of chemical attractants, e.g. 1-octen-3-ol, lactic acid, ammonia, [13,18,19] and means to increase humidity and temperature.

For *Anopheles gambiae sensu stricto *(henceforth termed *An. gambiae*), there is currently no combination of trapping device and bait available that can successfully compete with the human landing catch (HLC) as the standard method for population surveillance in the field [20]. Due to the possible exposure of field workers to infectious mosquito bites, cost, and tediousness of the HLC, this method poses both ethical and logistical problems (reviewed by [2]).

Recently, it was shown that rapid testing of candidate odour baits is possible in semi-field systems [21,22]. The partly controlled environment helps to yield statistically powerful results quickly in advance of full field evaluation, and it increases the potential to characterize mosquito responses to traps.

While there is no consensus on the exact role of CO_2 _in the behaviour of *An. gambiae *sensu lato, this compound is frequently used in trapping systems [16,17,23]. *Anopheles gambiae *responds strongly to combinations of human odour and CO_2 _[15] or human foot odour and CO_2 _[21,24]. This robust synergistic effect makes CO_2 _an important constituent of odour mixtures, although the practical value is limited due to technical and logistical problems under rural conditions [16].

Plant-derived essential oils can be used as mosquito repellents [25,26]. The repellency of plants themselves was surveyed during ethnobotanical studies in western Kenya by Seyoum *et al *[27], where traditional usage included direct burning of plant material and placement of repellent plants within houses. Initial experiments were conducted under semi-field conditions and later confirmed in field studies, where both potted plants and direct burning of *Corymbia citriodora, Ocimum kilimandscharicum *and *Ocimum suave *had a repellent effect. Using the latter method, the effect was comparable to commercial mosquito coils.

In this study, the trapping efficiency of two counterflow trap designs, the MM-X and BGS, was evaluated under semi-field conditions in Ifakara, Tanzania. Experimental baits included human foot odour and combinations of human foot odour with either carbon dioxide or lemongrass leaves.

## Methods

### Mosquitoes

*Anopheles gambiae *mosquitoes from an insectary colony maintained in Ifakara were used. This colony originates from Njage village, 80 km from Ifakara and has been reared under laboratory conditions since 1996. Eggs were collected on moist filter paper and transferred to trays for larval development. Larvae were kept at a density of about 500 individuals per tray in tap water and fed on Tetramin^® ^fish food. Room temperature was regulated to 30–32°C by an electric heating element. Pupae were collected daily and transferred to gauze-covered cages (30 × 30 × 30 cm) for hatching in a separate room (28–30°C, ca. 70% relative humidity). Adults were fed on 10% glucose solution, offered by placing soaked cotton wool on top of the cage. Blood feeding was done on the forearm of a human volunteer for 10 minutes 3, 7 and 10 days after emergence.

For all experiments, 200 unfed 2–5-day-old females were used. These were transferred into a small release cage (20 × 15 × 20 cm) 6 h prior to the experiments and only offered tap water from soaked cotton wool until the time of release.

### Experimental set-up

**Figure 1:**
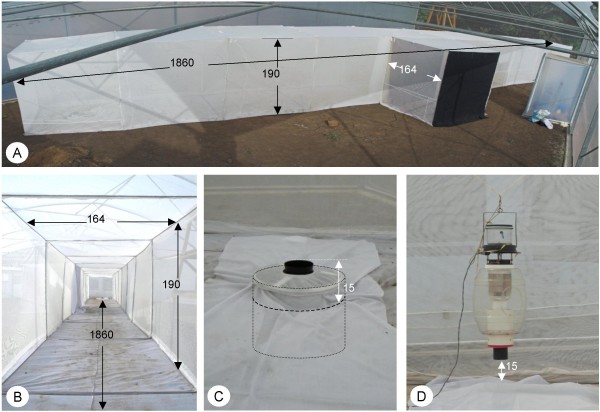
**Experimental set-up**. A: Panoramic photograph of the behavioural arena. B: Top and side view of the behavioural arena, showing exemplary placement of two BG Sentinel traps (black circle) and the position of the mosquito release cage (marked with X). C: The BG Sentinel trap, in the lowered position. D: The modified Mosquito Magnet-X trap, as used in the experiments.

A flight arena was constructed from locally available material. Eleven cubes (182 × 190 × 164 cm) were built from steel rods (Ø10 mm), painted and covered with a double layer of bednets and then sewn to a floor made of white cotton cloth. The experimental flight arena consisted of 10 of these elements, resulting in a total length of 18.3 m. One element was connected centrally to the side of the flight arena and used as an entrance chamber (Figures 1A and 1B). Two double-layer curtains sealed the entrance; the innermost layer consisted of white cloth, the other three curtains of heavy black cloth. The flight arena was placed diagonally in a 9.6 × 21 m large experimental compartment with a height ranging from 4 to 7 m. The floor consisted of a 35 cm thick layer of sand and earth on a concrete base. This compartment is part of a semi-field system recently completed at the Ifakara Health Research and Development Centre [28].

Temperature in the flight arena ranged from 22.1°C to 29.1°C (average 25.9°C, N = 64) at 7 pm, and from 17.4°C to 24.4°C (average 20.8°C, N = 64 nights) at 7 am. Relative humidity varied between 41.0% and 81.3% (average 59.2%) at 7 pm and between 60.0% and 86.4% (average 74.4%) at 7 am (measured with a TinyTag Ultra data logger, model TGU-1500, INTAB Benelux, Cuijk, The Netherlands).

### Traps

Traps were placed in the middle of the first and last segments, resulting in a distance of 16.5 m between them. Mosquito Magnet-X traps (MM-X, American Biophysics Corporation, modified as in [17], Figure 1D) were suspended from the top of the flight arena, with the odour outlet 15 cm above ground level. The bullet-shaped cartridge within the lower end of the odour outlet tube was removed in all experiments except when testing foot odour against clean socks when these were placed inside the cartridge.

The Sentinel traps (BGS, Biogents GmbH, Regensburg, Germany, Figure 1C) were placed below ground level, thereby also positioning the opening of this trap 15 cm above ground level. When no BGS was used, the opening in the ground was sealed with a wooden cover and white cloth. Both trap systems were connected to 12V car batteries, placed 3 m outside of the flight arena.

Cleaning procedures were adapted for both trap types. Ethanol (70%, Kas Medics, Tanzania) was used to clean all surfaces of the BGS trap and the odour outlet tube of the MM-X trap on a daily basis. Water and perfume-free soap (Neutral^® ^showergel, Intec B.V., Utrecht, The Netherlands) were used to clean the traps when the treatment was changed.

### Experimental procedures

Two hundred female mosquitoes were released each night in the centre of the flight arena at 19.00. At 07.00 the following morning, both traps were collected from the flight arena. Mosquitoes were killed by either placing the whole trap (MM-X) or the catching bag (BGS) in the sun and counted afterwards. Mosquitoes not caught during the night were left in the flight arena, where they died from exposure to sunlight during the day.

Treatments were tested for six consecutive nights, except the bait combinations foot odour with lemongrass or CO_2_, which were tested during four nights. During the experiments with unbaited traps and the direct comparison of a BGS against a MM-X trap, trap positions were randomized. For all other experiments, treatment positions were randomized each night to avoid positional effects.

### Odour cues

Foot odour was collected on nylon socks, worn for 12 hours by WHS prior to each experiment (07.00 to 19.00). For each experimental night, a recently-worn sock was used; a clean sock served as the control. In the MM-X trap, the sock was placed flat against the inside of the black central tube, thereby not obstructing the airflow. Further details and photos are shown in [12] and [21]. In the BGS trap, the sock was placed flat on a plastic bag and fixed to an aluminium dish (Ø 20 cm) at the bottom of the trap.

Carbon dioxide (Tanzania Oxygen Company, Tanzania) was available in pressurized gas cylinders and these were placed outside the flight arena. The gas flow was regulated by a manual flowmeter (Brooks Instruments, Veenendaal, The Netherlands) to 500 ml/min and supplied to the traps through silicone tubing (Ø 7 mm; Rubber B.V., Hilversum, The Netherlands). For the MM-X trap, the pre-installed plug was used to release the gas directly into the odour outlet. For the BGS trap the end of the tube was placed within the trap, fixed to the upper outer rim, pointing upwards.

Leaves of lemongrass (*Cymbopogon cf. citratus *– voucher: *Smallegange 2 *(WAG)) were collected 30 minutes prior to the experiments from potted plants received from north-western Tanzania. The plants were grown in a semi-shadowed place and watered daily. One gram of plant material was collected, cut into 5 cm long pieces, thoroughly bruised and placed within a worn sock. Care was taken to avoid unnecessarily blocking the air flow. Similarly treated grass leaves (*Stenotaphrum secundatum *– voucher: *Smallegange 1 *(WAG)), served as a control.

### Data analysis

All experiments were set up as binary choice tests. Catches were (n+1) transformed and analysed by logistic binary regression. By fitting the parameters position, experimental night and treatment to a generalized linear model of the form y = β_0 _+ β_1_x_1 _+ β_2_x_2 _the influence of the treatment was estimated [29]. All statistical analyses were done using SPSS version 11.5 (SPSS Inc., Chicago IL).

## Results

### Foot odour

Even in the absence of odour baits, mosquitoes were caught by both traps. While the average number of mosquitoes caught (± SD) in an unbaited MM-X trap was 6 ± 4, the unbaited BGS trap caught 41 ± 16 mosquitoes per night (Table 1). In both experiments, no significant differences could be found between the two individual MM-X (P = 0.36) or BGS (P = 0.36) traps.

**Table 1:** Competitive testing of two BGS or MM-X traps during 6 trap nights each, 200 *An. gambiae s.s. *females released per night.

Expt. A	Day	BGS unbaited	BGS unbaited	MM-X unbaited	MM-X unbaited
	1	62	43	10	5
	2	61	45	5	3
	3	31	33	12	12
	4	35	34	9	7
	5	43	11	2	0
	6	67	26	0	2
	Position	exp(B) = 0.365, P < 0.001		exp(B) = 1.137, P = 0.786	
	Treatment	exp(B) = 0.784, P = 0.358		exp(B) = 2.907, P = 0.358	
Expt. B	Day	BGS foot odour	BGS clean sock	MM-X foot odour	MM-X clean sock
	1	165	18	92	9
	2	170	14	61	5
	3	119	39	60	3
	4	172	21	55	2
	5	130	32	70	3
	6	145	29	41	7
	Position	exp(B) = 5.387, P < 0.001		exp(B) = 0.568, P = 0.348	
	Treatment	exp(B) = 0.135, P < 0.001		exp(B) = 0.206, P < 0.001	

A: Two unbaited traps, B: One trap baited with foot odour (worn nylon sock), one trap with a clean sock.

During initial experiments with the MM-X traps, socks worn for 12 hrs were placed loosely rolled in the bullet-shaped cartridge mounted in the odour outlet tube (Table 2). Over six nights, the average response with this set-up was 16 ± 4 for foot odour and 7 ± 2 for the control (P = 0.04). In all following experiments, the cartridge was removed and the socks were directly exposed to the airflow, by hanging them flat against the wall of the central tube. Thus MM-X traps baited with a worn sock caught 63 ± 17 mosquitoes compared to 5 ± 3 with a clean sock (P < 0.001). A similarly baited BGS trap caught 150 ± 22 mosquitoes compared to 26 ± 9 in the control trap (P < 0.001, Table 1).

**Table 2:** Competitive testing of two MM-X traps during 6 nights, one trap baited with foot odour (worn nylon sock in a bullet-shaped cartridge in the central tube), one trap with a clean sock.

Day	MM-X foot odour cartridge	MM-X clean sock cartridge
1	12	5
2	21	9
3	22	4
4	15	10
5	15	5
6	13	6
Position	exp(B) = 2.444, P = 0.030	
Treatment	exp(B) = 0.361, P = 0.040	

Each night 200 *An. gambiae s.s. *females were released.

**Figure 2:**
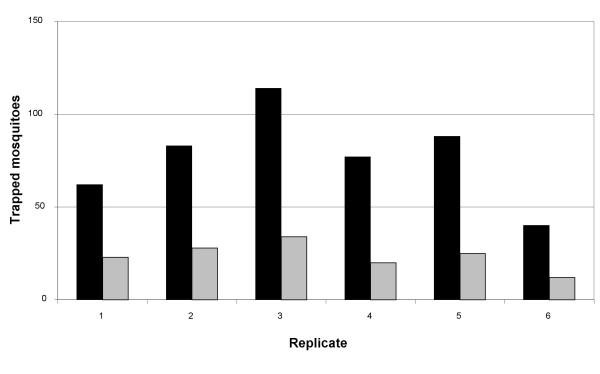
**Competitive trap tests with foot odour**. Mosquito catches of a BGS (black bars) and a MM-X (grey bars) trap running competitively for 6 nights. Both traps were baited with foot odour (nylon sock worn for 12 h by WHS), 200 *An. gambiae s.s. *females released per night.

In order to make a direct comparison between the two trapping systems, both trap types were baited with a worn nylon sock and tested for six nights (Figure 2). In line with the other data shown, the BGS trap (77 ± 25) caught on average about three times (P = 0.002) as many mosquitoes as the MM-X trap (24 ± 7).

### Odour combinations

Combinations of foot odour and 500 ml/min CO_2 _increased catches over foot odour alone in both types of traps, although this increase was not significant for the BGS trap. In the MM-X trap, on average 81 ± 23 mosquitoes responded to the combination with CO_2_, compared to 22 ± 4 to foot odour alone (P < 0.001). For the BGS trap, the combined bait led to the capture of 94 ± 14 mosquitoes versus 70 ± 12 with foot odour only (P = 0.051, Table 3).

**Table 3:** Competitive testing of two BGS or MM-X traps baited with A: foot odour (worn nylon sock in central tube) + CO_2 _(500 ml/min) or B: foot odour + lemongrass (1 g brushed leaves) during 4 nights each, 200 *An. gambiae s.s. *females released per night.

Expt. A	Day	BGS foot odour + CO_2_	BGS foot odour	MM-X foot odour + CO_2_	MM-X foot odour
	1	109	64	102	22
	2	100	84	98	16
	3	77	57	58	25
	4	90	75	64	24
	Position	exp(B) = 1.409, P = 0.032		exp(B) = 0.701, P = 0.218	
	Treatment	exp(B) = 0.714, P = 0.051		exp(B) = 0.422, P < 0.001	
Expt. B	Day	BGS foot odour + lemongrass	BGS foot odour + grass	MM-X foot odour + lemongrass	MM-X foot odour + grass
	1	72	78	14	34
	2	37	65	34	50
	3	53	68	33	40
	4	32	108	24	42
	Position	exp(B) = 0.488, P < 0.001		exp(B) = 2.380, P < 0.01	
	Treatment	exp(B) = 5.877, P < 0.001		exp(B) = 1.283, P = 0.353	

The addition of lemongrass to worn nylon socks led to a reduced catch rate in both trap types, although the result was only significant for the BGS trap. Here, the combination of foot odour and lemongrass caught 49 ± 18 mosquitoes compared to 80 ± 20 for foot odour alone (P < 0.001). When lemongrass leaves were added to a worn sock in a MM-X trap, 26 ± 9 mosquitoes were caught compared to 42 ± 7 for a worn sock only (P = 0.35, Table 3).

## Discussion

### Trap operation

Semi-field systems have already been suggested for high-throughput screening of candidate kairomones before [21,22]. Here it was shown that such systems can also be used to evaluate the trapping efficiency of two different counterflow traps.

The MM-X has been successfully used to capture anophelines in semi-field and field studies [11,17,21,23], while the BGS has mainly been developed for the collection of *Aedes aegypti *mosquitoes [13,14,30-32]. The BGS's collapsible, robust construction and its low weight make it highly portable and therefore more inviting for mosquito collections in the field compared to the MM-X. One notable feature of the BGS trap is its collection bag, which holds all trapped insects. It can be quickly replaced, and thereby offers the possibility to empty and restart a trap without the need of killing the catch on site.

During initial trials, the baited BGS trap was placed on the ground with the car batteries inside the flight arena. While this set-up successfully caught *Ae. aegypti *mosquitoes in the field [13,14], few *An. gambiae *females were found in the trap when baited with a worn sock (on average 29 ± 16, N = 2 nights). A surprisingly large number of mosquitoes was found resting and circling around the trap during the morning. After lowering the air intake relative to ground level by placing the trap in a pit, catch rates increased to the levels reported above. If similar behaviour is observed during field studies, this could restrict the usefulness of the trap in urban or rocky environments, when the trap cannot simply be placed below ground level.

In all experiments, catch rates for the unbaited BGS trap remained very high. Cleaning the traps with ethanol, water or perfume-free soap did not reduce the effect. It is thought that random dispersal effects or visual cues are the main reason for this observation. Pilot experiments in which the black air intake of one trap was covered by white paper suggest that mosquitoes respond stronger to a trap with a high-contrast trap entry. Due to the narrow construction of the flight arena, the larger radius of action of the suction capacity of the fan of the BGS trap compared to the MM-X trap (M. Geier, personal communication) might also facilitate trapping of not specifically attracted mosquitoes flying into the vicinity of the air intake. In addition, Biogents GmbH reports that the combination of the so-called 'forced upwards convection' (mimicking the convection currents created by the human body) and the downward suction forces makes the BGS trap superior compared to other traps when it comes to numbers of mosquitoes trapped (M. Geier, personal communication).

### Odour cues

The use of human foot odour collected on worn socks as a base attractant was chosen due to its known strong effect on *An. gambiae *females under laboratory [33,34], semi-field [21] and field conditions (S. Moore and M. Jawara, personal communication). The response level observed here is consistent with data reported for the MM-X trap under semi-field conditions by Njiru *et al *[21]. In all experiments with foot odour alone, however, the BGS trap achieved the highest catch rates.

The exact role of CO_2_in *An. gambiae *host-seeking behaviour remains unclear at present [16], although both our data and recent results by Njiru *et al *[21] and Spitzen *et al *[24] suggest a synergistic effect with human skin odours for host-seeking females. Despite the problems of using CO_2 _under field conditions, for example by the need of heavy gas tanks which need to be replaced frequently, it is widely used in different trap designs [2].

While the application of CO_2 _is straightforward with the MM-X trap, due to pre-installed plug connections, no similar mechanism was available for the BGS trap when we conducted our experiments. Releasing CO_2 _by simply pumping the gas (500 ml/min) to the bottom of the trap did not lead to increased catch rates as observed with the MM-X trap. As the release area of the odours is larger for BGS traps, this might be due to dilution effects. In addition, the effect of CO_2 _might be decreased when it is diluted by a convection like current instead of being released as a turbulent plume [24]. By releasing the gas at the outer rim of the trap, a non-significant increase could be observed, although at a lower level compared to the MM-X trap. Further experiments should focus on different positions of the gas valve and, possibly, varying the quantity of CO_2_.

Studies on the repellency of plant-derived essential oils [35-39], plant material [27,40] or potted plants [41] were published recently. Oil from *C. citratus *has been shown to have larvicidal activity against *Ae. aegypti *[42] and a repellent effect on *Ae. aegypti*, *Mansonia *spp. and *An. darlingi *[35,43]. While citronella oil, predominantly derived from *C. nardus *or *C. citratus *[44], is widely used in commercial insect repellents [45], lemongrass is one of several plant species currently planted in refugee camps in northern Tanzania and thought to repel mosquitoes and thereby lower the risk of mosquito-borne diseases. The experiments performed here show a reduced catch rate of 39% in the BGS trap, when offering leaves of lemon grass in combination with foot odour compared to foot odour alone. With the MM-X trap a similar catch reduction (of 37%) was observed, although this was not significant. It is possible that a higher quantity of lemongrass would have shown a larger effect with both trap types. First and foremost, our results show the possibility to evaluate samples of plant material and putative repellent compounds in the described system. It also appears to be worthwhile to further explore the potential and optimal application strategy of lemongrass as a repellent for *An. gambiae*. When testing repellent plant material with mosquito traps, the limiting factor is the required number of mosquitoes caught to achieve significant results. This implies the usage of the strongest available bait in combination with a trap design able to offer high discriminatory power within a contained system.

## Conclusion

Within the described set-up, the two different counter-flow traps demonstrated markedly distinct results. The BGS trap showed a consistently higher catching efficiency for *An. gambiae *than the MM-X trap, especially when using human odours or no bait at all. While this behaviour might be attributed to the characteristics of the specific behavioural arena, it also suggests that the BGS trap may operate very well under field conditions, even without additional CO_2 _if necessary. The robust construction and the high portability further recommend the BGS trap for field trials as a sampling tool for *An. gambiae*.

The results with combined baits demonstrate that the semi-field system used in our experiments can be used for rapid screening of both synthetic and natural odour baits. For this purpose, the higher discriminatory power of the MM-X trap appears advantageous.

## Competing interests

The authors declare that they have no competing interests.

## Authors' contributions

The initial experimental set-up was developed by RCS, WT and GFK. WHS conducted the experiments and drafted the manuscript with RCS. GFK assisted with the statistical analyses. WT and BGJK provided technical advice and contributed to drafting the final manuscript. All authors approved the final version of the manuscript.

## References

[B1] Service MW (1993). Mosquito Ecology: field sampling methods.

[B2] Qiu YT, Spitzen J, Smallegange RC, Knols BGJ, Takken W, Knols BGJ (2007). Monitoring systems for adult insect pests and disease vectors. Emerging pests and vector-borne diseases in Europe.

[B3] Sudia WD, Chamberlain RW (1988). Battery-operated light trap, an improved model. By W. D. Sudia and R. W. Chamberlain, 1962. J Am Mosq Control Assoc.

[B4] Costantini C, Gibson G, Brady J, Merzagora L, Coluzzi M (1993). A new odour-baited trap to collect host-seeking mosquitoes. Parassitologia.

[B5] Dia I, Diallo D, Duchemin JB, Ba Y, Konate L, Costantini C, Diallo M (2005). Comparisons of human-landing catches and odor-baited entry traps for sampling malaria vectors in Senegal. J Med Entomol.

[B6] Mathenge EM, Killeen GF, Oulo DO, Irungu LW, Ndegwa PN, Knols BGJ (2002). Development of an exposure-free bednet trap for sampling Afrotropical malaria vectors. Med Vet Entomol.

[B7] Mathenge EM, Misiani GO, Oulo DO, Irungu LW, Ndegwa PN, Smith TA, Killeen GF, Knols BGJ (2005). Comparative performance of the Mbita trap, CDC light trap and the human landing catch in the sampling of *Anopheles arabiensis*, *An. funestus *and culicine species in a rice irrigation in western Kenya. Malar J.

[B8] Mathenge EM, Omweri GO, Irungu LW, Ndegwa PN, Walczak E, Smith TA, Killeen GF, Knols BGJ (2004). Comparative field evaluation of the Mbita trap, the Centers for Disease Control light trap, and the human landing catch for sampling of malaria vectors in western Kenya. Am J Trop Med Hyg.

[B9] Knols BGJ, Mboera LE, Takken W (1998). Electric nets for studying odour-mediated host-seeking behaviour of mosquitoes. Med Vet Entomol.

[B10] Cooperband MF, Carde RT (2006). Orientation of Culex mosquitoes to carbon dioxide-baited traps: flight manoeuvres and trapping efficiency. Med Vet Entomol.

[B11] Kline DL (1999). Comparison of two American biophysics mosquito traps: the professional and a new counterflow geometry trap. J Am Mosq Control Assoc.

[B12] Kline DL (2002). Evaluation of various models of propane-powered mosquito traps. J Vector Ecol.

[B13] Kröckel U, Rose A, Eiras ÁE, Geier M (2006). New tools for surveillance of adult yellow fever mosquitoes: comparison of trap catches with human landing rates in an urban environment. J Am Mosq Control Assoc.

[B14] Maciel-de-Freitas R, Eiras ÁE, Lourenco-de-Oliveira R (2006). Field evaluation of effectiveness of the BG-Sentinel, a new trap for capturing adult *Aedes aegypti *(Diptera: Culicidae). Mem Inst Oswaldo Cruz.

[B15] Costantini C, Gibson G, Sagnon N, Della Torre A, Brady J, Coluzzi M (1996). Mosquito responses to carbon dioxide in a west African Sudan savanna village. Med Vet Entomol.

[B16] Mboera LEG, Takken W (1997). Carbon dioxide chemotropism in mosquitoes (Diptera: Culicidae) and its potential in vector surveillance and management programmes. Rev Med Vet Entomol.

[B17] Qiu YT, Smallegange RC, Ter Braak CJF, Spitzen J, Van Loon JJA, Jawara M, Milligan P, Galimard AM, Van Beek TA, Knols BGJ, Takken W (2007). Attractiveness of MM-X traps baited with human or synthetic odor to mosquitoes (Diptera: Culicidae) in The Gambia. J Med Entomol.

[B18] Takken W (1996). Synthesis and future challenges: the response of mosquitoes to host odours. Ciba Foundation Symposium.

[B19] Takken W, Knols BGJ (1999). Odor-mediated behavior of Afrotropical malaria mosquitoes. Annu Rev Entomol.

[B20] Mboera LEG (2005). Sampling techniques for adult Afrotropical malaria vectors and their reliability in the estimation of entomological inoculation rate. Tanzan Health Res Bull.

[B21] Njiru BN, Mukabana WR, Takken W, Knols BGJ (2006). Trapping of the malaria vector *Anopheles gambiae *with odour-baited MM-X traps in semi-field conditions in western Kenya. Malar J.

[B22] Knols BGJ, Njiru BN, Mathenge EM, Mukabana WR, Beier JC, Killeen GF (2002). MalariaSphere: a greenhouse-enclosed simulation of a natural *Anopheles gambiae *(Diptera: Culicidae) ecosystem in western Kenya. Malar J.

[B23] Mboera LEG, Knols BGJ, Braks MAH, Takken W (2000). Comparison of carbon dioxide-baited trapping systems for sampling outdoor mosquito populations in Tanzania. Med Vet Entomol.

[B24] Spitzen J, Smallegange RC, Takken W (2008). Effect of human odours and positioning of CO2 release point on trap catches of the malaria mosquito *Anopheles gambiae sensu stricto *in an olfactometer. Physiol Entomol.

[B25] Ansari MA, Razdan RK (1995). Relative efficacy of various oils in repelling mosquitoes. Indian J Malariol.

[B26] Odalo JO, Omolo MO, Malebo H, Angira J, Njeru PM, Ndiege IO, Hassanali A (2005). Repellency of essential oils of some plants from the Kenyan coast against *Anopheles gambiae*. Acta Trop.

[B27] Seyoum A, Palsson K, Kung'a S, Kabiru EW, Lwande W, Killeen GF, Hassanali A, Knols BGJ (2002). Traditional use of mosquito-repellent plants in western Kenya and their evaluation in semi-field experimental huts against *Anopheles gambiae*: ethnobotanical studies and application by thermal expulsion and direct burning. Trans R Soc Trop Med Hyg.

[B28] Ferguson HM, Ng'habi KR, Walder T, Kadungula D, Moore SJ, Lyimo I, Russell TL, Urassa H, H Mshinda, Killeen GF, Knols BGJ (2008). Establishment of a large semi-field system for experimental study of African malaria vector ecology and control in Tanzania. Malar J.

[B29] Collett D (2003). Modelling Binary Data.

[B30] Williams CR, Bergbauer R, Geier M, Kline DL, Bernier UR, Russell RC, Ritchie SA (2006). Laboratory and field assessment of some kairomone blends for host-seeking *Aedes aegypti*. J Am Mosq Control Assoc.

[B31] Williams CR, Long SA, Russell RC, Ritchie SA (2006). Field efficacy of the BG-Sentinel compared with CDC Backpack Aspirators and CO2-baited EVS traps for collection of adult *Aedes aegypti *in Cairns, Queensland, Australia. J Am Mosq Control Assoc.

[B32] Williams CR, Long SA, Webb CE, Bitzhenner M, Geier M, Russell RC, Ritchie SA (2007). *Aedes aegypti *population sampling using BG-Sentinel traps in north Queensland Australia: statistical considerations for trap deployment and sampling strategy. J Med Entomol.

[B33] De Jong R, Knols BGJ (1995). Selection of biting sites on man by two malaria mosquito species. Experientia.

[B34] Qiu YT, Smallegange RC, Smid H, Van Loon JJA, Galimard AMS, Posthumus MA, Van Beek TA, Takken W (2004). GC-EAG analysis of human odours that attract the malaria mosquito *Anopheles gambiae sensu stricto*. Proc Exp Appl Entomol, NEV; Groningen.

[B35] Moore SJ, Hill N, Ruiz C, Cameron MM (2007). Field Evaluation of Traditionally Used Plant-Based Insect Repellents and Fumigants Against the Malaria Vector *Anopheles darlingi *in Riberalta, Bolivian Amazon. J Med Entomol.

[B36] Tawatsin A, Wratten SD, Scott RR, Thavara U, Techadamrongsin Y (2001). Repellency of volatile oils from plants against three mosquito vectors. J Vector Ecol.

[B37] Fradin MS, Day JF (2002). Comparative efficacy of insect repellents against mosquito bites. N Engl J Med.

[B38] Trongtokit Y, Rongsriyam Y, Komalamisra N, Apiwathnasorn C (2005). Comparative repellency of 38 essential oils against mosquito bites. Phytother Res.

[B39] Moore SJ, Darling ST, Sihuincha M, Padilla N, Devine GJ (2007). A low-cost repellent for malaria vectors in the Americas: results of two field trials in Guatemala and Peru. Malar J.

[B40] Seyoum A, Killeen GF, Kabiru EW, Knols BGJ, Hassanali A (2003). Field efficacy of thermally expelled or live potted repellent plants against African malaria vectors in western Kenya. Trop Med Int Health.

[B41] Seyoum A, Kabiru EW, Lwande W, Killeen GF, Hassanali A, Knols BGJ (2002). Repellency of live potted plants against *Anopheles gambiae *from human baits in semi-field experimental huts. Am J Trop Med Hyg.

[B42] Cavalcanti ESB, De Morais SM, Lima MAA, Santana EWP (2004). Larvicidal activity of essential oils from Brazilian plants against *Aedes aegypti *L. Mem Inst Oswaldo Cruz.

[B43] Oyedele AO, Gbolade AA, Sosan MB, Adewoyin FB, Soyelu OL, Orafidiya OO (2002). Formulation of an effective mosquito-repellent topical product from Lemongrass oil. Phytomed.

[B44] Verhulst NO, Curtis CF, Hill N, Takken W, Knols BGJ (2007). Personal protection against European disease vectors. Emerging pests and vector-borne diseases in Europe.

[B45] Barnard DR, Xue RD (2004). Laboratory evaluation of mosquito repellents against *Aedes albopictus*, *Culex nigripalpus*, and *Ochierotatus triseriatus *(Diptera: Culicidae). J Med Entomol.

